# Influence of socioeconomic variables on physical activity and screen time of children and adolescents during the COVID‑19 lockdown in Germany: the MoMo study

**DOI:** 10.1007/s12662-021-00783-x

**Published:** 2021-12-03

**Authors:** Steffen C. E. Schmidt, Alexander Burchartz, Simon Kolb, Claudia Niessner, Doris Oriwol, Alexander Woll

**Affiliations:** 1 Karlsruhe Institute of Technology, Engler-Bunte-Ring 15, 76131 Karlsruhe, Germany; 2 University of Education Karlsruhe, Bismarckstraße 10, 76133 Karlsruhe, Germany

**Keywords:** Outdoor play, Sports, Youth, Socioeconomic status, MoMo

## Abstract

The COVID‑19 (coronavirus disease 2019) pandemic created a multitude of natural experiments about the change of human behavior in a widely unfamiliar situation. Besides physical and mental health, physical activity (PA) and people’s movement behaviors were of particular interest to researchers all over the world. In a recent study, we found that among youth in Germany, sports activity declined, whereas recreational screen time and habitual activity increased during the first COVID‑19 lockdown. In the present study, we analyze the influence of the socioeconomic status and the housing situation on the changes in PA behavior and recreational screen-time before and during the first COVID‑19 lockdown among children and adolescents living in Germany. We found an alignment of PA behavior among youth from families with different socioeconomic backgrounds during the first lockdown and identified the housing situation to be a meaningful predictor of the increase in habitual activity. We conclude that restriction policies, communities, and in the last instance parents need to enable access to nonorganized PA to all children and adolescents every day and especially during potential future lockdowns.

## Introduction

An active lifestyle during childhood has been associated with improved physical and mental health (Ekelund et al., 2016; Warburton & Bredin, 2017) and reduced risk for several diseases in adulthood (Janssen, & LeBlanc, 2010; Twisk, Kemper, & van Mechelen, 2002). With digitalization being one of the megatrends of the 21st century, the ideal phenotype of a healthy balance between sedentary screen-time (ST) and physical activity (PA) is moving more and more to the center of epidemiological interests, especially when youth are concerned (Viner, Davie, & Firth, 2019; Elson et al., 2019; Viner et al., 2019; Ashton & Beattie, 2019). In the process, the World Health Organization (WHO) recently updated their PA and ST guidelines to an average of 60 min of moderate-to-vigorous daily PA for children and adolescents and to limit the amount of recreational screen time (DiPietro et al., 2020; Bull et al., 2020).

PA can take place in different settings with different underlying motives. In Germany and most other western countries, organized forms of PA that take place in sports clubs and schools form a significant proportion of regular, periodic PA (Tremblay et al., 2016; Barlow, 2007; Schmidt et al., 2020a). It is also know that healthy behaviors are complied more successful on structured compared to unstructured days (Brazendale et al., 2017). Therefore, the shutdown of organized sports and public sports facilities in most countries at the beginning of the coronavirus disease 2019 (COVID‑19) outbreak resulted in crucial changes in youth’s daily routines and their PA opportunities. In Germany, the federal states closed kindergartens, schools, sports clubs, gyms, and other leisure institutions relevant to children’s and adolescents’ organized PA from 16–18 March 2020 until 3 May 2020. The government also imposed physical distancing measures and contact restrictions, allowing no more than two people from different households to meet in public spaces (Press and Information Office of the Federal Government, 2020a). However, in Germany, nonorganized PA such as workouts at home and other forms of habitual PA besides sports (HA), like playing outside remained allowed if done alone or with people from the same household (Press and Information Office of the Federal Government, 2020b).

Many researchers assumed that COVID‑19 reinforces sedentariness due to missing PA opportunities and physical distancing (Rundle, Park, Herbstman, Kinsey, & Wang, 2020; Hall, Laddu, Phillips, Lavie, & Arena, 2020; Fegert, Vitiello, Plener, & Clemens, 2020; Xiang, Zhang & Kuwahara, 2020). Studies from all over the world such as Canada (Hemphill, Kuan, & Harris, 2020; Guerrero et al., 2020; Moore et al., 2020), China (Xiang et al., 2020; Zenic et al., 2020), Spain (López-Bueno et al., 2020), Italy (Pietrobelli et al., 2020), and the US (Dunton, Do, & Wang, 2020) proved them right when using retrospective questionnaires or device-based measurements. In most countries and also when looking at aggregated device-based data (Garmin, 2020; Tison et al., 2020), PA among youth has declined during the COVID‑19 pandemic.

To analyze the situation during the first lockdown in Germany, we surveyed the participants of the third wave (2018–2020) of the Motorik-Modul Study (MoMo), using an online version of the MoMo physical activity questionnaire (Schmidt, Will, Henn, Reimers, & Woll, 2016). This approach allows us to directly compare the PA of a representative drawn sample of youth living in Germany before (MoMo wave 3, pre-study) and during the pandemic (MoMo first lockdown survey, peri study). We decided to disseminate our main results through two papers. The first paper is targeting an international audience, focusing on the shifts in PA behavior among different PA domains, stratified by age and sex to allow comparisons with studies from other countries (Schmidt et al., 2020b). The present work reflects the second paper which in turn focuses on socioeconomic context factors by stratifying the sample by socioeconomic status (SES) and different environmental factors to discuss effects in the context of specific vulnerable groups within Germany. The data we present in the first paper showed a more sophisticated picture of PA behavior compared to most international studies, especially when looking at both sports activity (SA) and HA (Schmidt et al., 2020b). We found that SA declined, whereas ST and HA increased among children and adolescents irrespective of sex and age (Schmidt et al., 2020b). Youth in Germany successfully transferred their structured PA to unstructured, at least for the brief moment of the first lockdown. This led to an overall increase of PA among youth during the first COVID‑19 lockdown in Germany. The underlying factors for this behavior in contrast to other countries are yet unknown, but we suggest them to be (a) different restrictions by policy, (b) the number of COVID‑19 infections, (c) more time for recreational activities (d) self-determination theory effects combined with a more pronounced focus on health, and (e) different methodological quality and data-evaluation approaches of the studies (Schmidt et al., 2020b). For example, a study from Croatia collected data from 823 adolescents and showed that PA levels decreased primarily in adolescents living in urban areas (Zenic et al., 2020). A recent narrative review also confirms the suspicion of socioeconomic differences in health behavior during the COVID‑19 pandemic for adults (Jordan et al., 2020).

Currently we do not know to what degree the changes in PA behavior are stable, i.e., have transferable effects and whether there are different patterns of behavior according to socioeconomic context factors such as SES and environment. In the following, we analyze the PA and ST data of a nationwide sample of youth aged 6‑to-17 prior to (pre) and during (peri) the first COVID‑19 lockdown stratified for different socioeconomic factors. Thereby, we investigate how SA, HA, and recreational ST changed among youth from families with different SES and different housing situations during the first COVID‑19 lockdown.

## Methods

All data were obtained within the framework of the MoMo study (Woll et al., 2021). The study follows a cohort sequence design to analyze and track the physical activity, fitness, and health of children and adolescents living in Germany. In addition to a longitudinal sample of children and adolescents who were recruited between 2003 and 2012, a new representative cross-sectional sample of 4‑ to 17-year-old children and adolescents living in Germany is recruited at each follow-up (Woll et al., 2021). Wave 3 started in August 2018 and was planned to be finished in June 2020, but had to be interrupted in March 2020 because of the first COVID‑19 lockdown in Germany. Within the theoretical framework of a natural experiment, we refer to this data as the pre-study. For the peri-study, we asked every participant of Wave 3 hitherto to answer the questionnaire again, but this time during the strictest interval of the first lockdown in Germany (20 April 2020–01 May 2020). Detailed information about the sequence of events can be found elsewhere (Schmidt, et al., 2020b).

### Participants and procedure

For the pre-study, parents and children were invited to examination rooms at central locations in the proximity of their homes. Parents gave their written consent for minors and the presence of a legal guardian was mandatory under the age of 15. Questionnaires were filled out on-site by the participants on laptops (pre) or online (peri). Participation in the study was voluntary and every participant received a gift (value 10–20 €). The participants or their custodians were informed about the contents of the study and data protection and gave their written consent. For the peri-study, the participants were contacted via email, reminded up to two times, and asked to answer the peri-questions online.

All initial Wave 3 participants were selected based on a nationwide multistage sampling approach with two evaluation levels (Kamtsiuris, Lange & Schaffrath-Rosario, 2007) to maximize representativeness. First, a systematic sample of 167 primary sampling units was selected from an inventory of German communities stratified according to the classification system that measures the level of urbanization and geographic distribution (Kurth et al., 2008). The probability of any community being picked was proportional to the number of inhabitants younger than 18 years of age in that community. Second, based on the official registers of residents, an age-stratified sample of randomly selected children and adolescents was drawn.

In response to the COVID‑19 situation in Germany, Wave 3 had to be interrupted in March of 2020 after 114 of 167 locations were visited. In total, 2722 MoMo Wave 3 participants (initial response: 25.2%) were contacted for the peri-study, and data from 1711 participants were gathered (longitudinal response: 63.6%). Twenty-three e‑mails could not be delivered because of incorrect addresses. We decided to exclude the 4‑ and 5‑year-old participants in the current paper because we think that their PA behavior, motivation, and influencing factors differ from the more autonomous 6–17 year olds and an age-stratified discussion of each socioeconomic context factor would go beyond the scope of this paper. Therefore, please refer to our first paper (Schmidt et al., 2020b) for data among 4–5 year olds living in Germany.

A total of 1394 data sets remained for the analysis. Of those, 1387 produced evaluable data on relevant socioeconomic variables, 1345 on PA, and 1360 on ST. To differentiate between youth in the primary and secondary school age, we decided to report the results stratified by two age groups: 6–10 and 11–17. Table 1 shows the characteristics of the longitudinal sample.

**Table 1:** Sample characteristics

Age group	*n*	Age pre M ± s (years)	Sex (% female)	Socioeconomic status		
				Low (%)	Mid (%)	High (%)
6 to 10 years	647	8.3 ± 1.4	46.7	14.3	57.5	28.3
11 to 17 years	747	14.4 ± 2.0	53.9	18.6	59.7	21.7
All participants	1394	11.6 ± 3.5	50.5	16.6	58.6	24.8

*M* mean, *s* standard deviation

### Measures

During the pre-study, data about PA and ST were derived via questionnaires and socioeconomic data via interviews on the pursuant screening site. For children under the age of 11 years, parents were asked to fill in the questionnaires together with the child. This was done in 93.9% of the cases pre and 94.0% of the cases peri. During the peri-study, the identical questions about PA and ST were asked via an online version of the questionnaire except for the questions about organized PA which had been prohibited by governmental law. We also changed each instance of time mentioned in the description of the questions during the peri-study to “during the Corona lockdown”. Among both age groups, 38.2% of the peri-study online questionnaires were filled out by the youth alone, 49.8 together with the mother, 9.8 with the father, and 2.3 with another person.

*Physical activity:* The MoMo PA Questionnaire (MoMo-PAQ) was used to assess PA via self-reported SA and HA in the settings of sports clubs, leisure time, and school (Schmidt et al., 2016). Originating from the definition of PA as “any bodily movement produced by skeletal muscles that result in energy expenditure” (Casperson, Powell, & Christenson, 1985), we refer to SA as any PA that is accumulated by sports and to HA as any PA that is accumulated by daily PA which is not linked to sports. In the MoMo study, this definition of HA is operationalized through playing outside, gardening, housework, as well as walking and cycling (or similar activities like riding a non-motorized scooter) for transportation.

The MoMo physical activity questionnaire follows the consensus of assessing PA through questionnaires (Nigg et al., 2020). The data obtained with the MoMo-PAQ are sufficiently reliable and valid (test–retest reliability: ICC = 0.68) (Jekauc, Wagner, Kahlert, & Woll, 2013). SA at school was assessed by two items about the frequency (times per week) of 45 min classes in curricular and extracurricular sports activities which are multiplied by a factor of 8.5/12 to correct for vacations. SA in sports clubs was assessed by two items which could be answered up to four times: type of sports club activity and duration (minutes per week). During the pre-study, we also asked on which specific month throughout the year the sport is done. Minutes were then multiplied by the number of months per year divided by twelve to control for periodic effects of SA. Nonorganized leisure-time SA was assessed in the same way by three items and could be answered for up to four types of sports. All instances of SA were then combined in an index that reflects the daily minutes with SA (total amount of sports). Types of sports that do not lead to an increase in energy expenditure using large skeletal muscles (for example eSports or chess) were not defined as SA (Caspersen et al., 1985).

Nonorganized playing outside, gardening, and housework were each assessed separately by two items about days per week and minutes a day in which the participants pursued the activity (“On how many days do you normally play outside/garden/work in the household during a week?”, “How long do you on average work/play on one of those days?”). Walking and cycling were assessed by one item each about the daily distance and time participants travel by walking or cycling (Schmidt et al., 2016). Minutes spent playing outside, walking, cycling, gardening, and housework were then combined in an index that reflects the daily minutes of HA (total amount of habitual activity).

*Recreational screen time:* ST was measured via self-reported screen-time behaviors which are commonly used to report ST in youth and which have similar directions with health outcomes as direct measures of sedentary behavior (Tremblay et al., 2011). Participants were asked to report the time spent watching television (TV), playing games on any device (Gaming), and using the internet for recreational use (Internet) separately for weekdays and weekends using an 8‑point scale including (almost) never, 15 min per day, 30 min per day, 1 h per day, 2 h per day, 3 h per day, and 4 h per day (Mathers et al., 2009). An index reflecting weekdays and weekend days at a 5:2 ratio was then calculated (total amount of recreational screen-time).

*Sociodemographic variables:* Sociodemographic variables were assessed within the pre-study, including age, sex, and a multitude of socioenvironmental and socioeconomic variables. Individual-level SES was defined according to the educational and professional status of the parents as well as the total household income per household member. Education and professional status were asked separately for both parents with the higher score being used (Lampert, Müters, Stolzenberg, & Kroll, 2014). Adolescents with separated parents were assigned the socioeconomic status of the parent they lived with. The three aspects income, educational status, and professional status were scored on a scale from 1 to 7 and a sum score was created (range: 3–21). The sum score was then categorized by quintiles of the whole MoMo Wave 3 sample into low (first quintile: 3–12), medium (12.1–18.5), and high (fifth quintile: 18.6–21) SES. Detailed information about this approach can be found elsewhere (Winkler & Stolzenberg, 2009; Lampert et al., 2014; Lampert, Hoebel, Kuntz, Müters, & Kroll, 2018). The housing situation was assessed during the peri-study with an item about the characteristics of the property (detached house, semi-detached house, multifamily home, multifamily home with more than six parties) and one dichotomous item about the access to a garden/yard owned by the family.

*PA and ST guideline adherence: *Compliance with PA recommendations of the WHO was defined following the 2011 WHO PA recommendations of 60 min of MVPA on each day (WHO, 2011). We asked the participants on how many days they are active for more than 60 min with moderate to vigorous intensity to reproduce the guideline in accordance to Prochaska, Sallis, and Long (2001): “Over a typical or usual week (peri: ‘during the Corona lockdown’), on how many days are you physically active for a total of at least 60 min per day?”. Although there is no consensus yet for ST recommendations in children and adolescents, guideline adherence was defined according to recent recommendations (Tremblay et al., 2016) as to whether or not the average daily recreational ST exceeded 120 min.

### Statistics

All statistical tests were conducted using IBM SPSS 26 (IBM Corporation, Armonk, NY, USA). Statistically significant (two-sided) differences pre and peri were determined via analysis of variance (ANOVA) for repeated measurements with sex and SES as factors (between) where the effect of time represents the lockdown effect (within). Analyzes for the total amount of SA, HA, and ST were run separately for 6–10 and 11–17 year olds. Further analyzes of the impact of different socioeconomic variables on different types of HA were run as one model including all between-factors at once with the type of HA pre and peri being the dependent variable (Table 3). For all analyses, cases with missing values concerning SA, HA, and ST for either pre or peri lockdown were deleted listwise within the respective ANOVA. That means, only participants with full information were included. The number of missing cases can be derived from reported degrees of freedom for the related f-values. To enable inference by eye, 95% confidence intervals (95% CI) for differences of units and proportions (Cumming & Fidler, 2009) are reported. Whenever a reported 95% CI for differences includes zero, i.e., 0.0, *p* is larger than 0.05 and the difference is not significant in the considered sample (Cumming & Fidler, 2009).

## Results

Table 2 shows the total amount of sports and habitual activity pre and peri lockdown in Germany stratified by age group, sex, and SES, as well as the compliance with PA recommendations of the WHO (WHO, 2011).

**Table 2:** Physical activity and guideline adherence stratified by socioeconomic status before and during the COVID‑19 lockdown in Germany (MoMo study)

			Total amount of sports activity (minutes per day)			Total amount of habitual activity (minutes per day)			PA guideline adherence (%)		
**Age 6–10**		***N***	**Pre** **(M** **±** **s)**	**Peri** **(M** **±** **s)**	**Peri–pre** **(diff. (95% CI))**	**Pre** **(M** **±** **s)**	**Peri** **(M** **±** **s)**	**Peri–pre** **(diff. (95% CI))**	**Pre**	**Peri**	**Peri–pre** **(diff. (95% CI))**
Low	m	44	34.2 ± 39.7	8.5 ± 23.1	−25.7 (−38.3, −13.0)**	135.8 ± 100.1	151.9 ± 140.2	+16.0 (−17.4, 49.4) n. s.	29.5	35.6	+6.0 (−13.2, 24.5) n. s*.*
	f	43	23.1 ± 15.7	11.7 ± 26.1	−11.4 (−19.2, −3.6)**	130.0 ± 103.7	184.1 ± 133.0	+54.0 (12.8, 95.3)*	13.3	34.8	+21.5 (3.8, 37.5)*
	Ø	87	28.7 ± 30.6	10.1 ± 24.6	−18.6 (−26.1, −11.1)**	132.8 ± 101.4	168.5 ± 136.7	+35.7 (9.2, 61.7)**	21.3	35.2	+13.8 (0.6, 26.3)*
Mid	m	198	34.6 ± 22.5	26.6 ± 40.1	−7.9 (−14.0, −1.9)*	121.2 ± 71.6	174.2 ± 108.2	+53.0 (37.6, 68.4)**	30.5	47.1	+16.5 (7.1, 25.6)*
	f	155	28.4 ± 16.2	20.4 ± 33.4	−8.0 (−13.4, −2.6)**	118.2 ± 79.5	165.8 ± 108.4	+47.7 (31.5, 63.7)**	26.1	37.0	+11.0 (0.8, 20.8)*
	Ø	353	31.9 ± 20.2	23.9 ± 37.4	−8.0 (−12.1, −3.9)**	119.9 ± 75.1	170.5 ± 108.2	+50.6 (39.5, 61.7)**	28.6	42.6	+14.1 (7.1, 20.8)*
High	m	90	41.6 ± 22.3	29.2 ± 39.9	−12.3 (−21.2, −3.6)**	120.5 ± 60.6	167.7 ± 105.1	+47.2 (26.0, 68.3)**	25.3	41.3	+16.0 (2.4, 28.9)*
	f	87	36.1 ± 17.9	24.8 ± 34.0	−11.3 (−18.7, −3.9)**	106.9 ± 55.0	151.7 ± 99.5	+44.8 (26.7, 62.9)**	21.6	40.9	+19.3 (5.6, 32.1)*
	Ø	177	38.9 ± 20.4	27.0 ± 37.1	−11.9 (−17.6, −6.2)**	114.0 ± 58.2	160.0 ± 102.5	+46.0 (32.1, 59.9)**	23.5	41.1	+17.6 (8.0, 26.9)*
All	m	336	36.5 ± 25.5	24.7 ± 38.5	−15.0 (−5.0, −25.1)**	122.7 ± 72.7	169.1 ± 111.3	+46.6 (34.9, 58.0)**	28.7	44.1	+15.4 (8.2, 22.4)*
	f	291	30.3 ± 18.2	20.5 ± 32.8	−10.1 (−0.6, −19.5)**	116.2 ± 77.3	163.4 ± 109.5	+47.2 (35.4, 58.9)**	22.7	37.4	+14.8 (7.4, 21.9)*
	Ø	627	33.6 ± 22.6	22.8 ± 36.0	−10.8 (−7.8, −13.8)**	119.6 ± 74.9	166.4 ± 110.4	+46.8 (38.6, 55.0)**	25.9	41.0	+15.1 (10.0, 20.1)*
**Age 11–17**		***N***	**Pre** **(M** **±** **s)**	**Peri** **(M** **±** **s)**	**Peri–pre** **(diff. (95% CI))**	**Pre** **(M** **±** **s)**	**Peri** **(M** **±** **s)**	**Peri–pre** **(diff. (95% CI))**	**Pre**	**Peri**	**Peri–pre** **(diff. (95% CI))**
Low	m	63	37.6 ± 23.6	25.0 ± 36.9	−12.6 (−22.7, −2.5)*	100.7 ± 82.8	118.3 ± 103.6	+17.7 (−6.0, 41.3) *n.* *s.*	11.1	25.4	+14.3 (0.7, 27.5)*
	f	66	31.3 ± 27.0	24.7 ± 35.1	−6.6 (−15.1, +1.9) *n.* *s.*	85.5 ± 85.7	114.0 ± 114.9	+28.6 (3.3, 53.9)*	10.0	8.5	−1.5 (−11.8, 8.6) *n.* *s.*
	Ø	129	34.4 ± 25.5	24.8 ± 35.8	−9.5 (−16.0, −3.1)**	92.7 ± 84.3	116.1 ± 109.3	+23.4 (6.2, 40.5)**	10.5	16.5	+5.9 (−2.4, 14.2) *n.* *s.*
Mid	m	188	43.4 ± 24.7	24.2 ± 37.7	−19.2 (−24.1, −14.3)**	94.8 ± 73.4	101.3 ± 97.3	+6.5 (−6.3, 19.2) *n.* *s.*	8.8	12.3	+3.5 (−2.7, 9.8) *n.* *s.*
	f	225	41.8 ± 26.1	28.2 ± 34.2	−13.6 (−18.5, −8.8)**	74.4 ± 47.8	96.1 ± 76.9	+21.7 (12.5, 30.9)**	5.6	11.1	+5.6 (0.5, 10.8)*
	Ø	413	42.5 ± 25.4	26.4 ± 35.8	−16.2 (−19.6, −12.7)**	83.7 ± 61.6	98.5 ± 86.7	+14.8 (7.1, 22.4)**	7.0	11.7	+4.6 (0.7, 8.6)*
High	m	69	60.3 ± 33.1	49.5 ± 51.4	−10.8 (−23.9, +2.4) *n.* *s.*	88.0 ± 66.3	124.6 ± 101.5	+36.6 (12.0, 61.2)**	13.9	23.6	+9.7 (−3.1, 22.3) *n.* *s.*
	f	81	52.9 ± 35.0	37.4 ± 32.5	−15.5 (−24.1, −7.0)**	76.2 ± 48.9	91.8 ± 55.7	+15.6 (3.2, 28.0)*	6.0	15.5	+9.5 (−0.1, 19.3) *n.* *s.*
	Ø	150	56.3 ± 34.3	42.9 ± 42.5	−13.3 (−20.9, −5.8)**	81.7 ± 57.7	107.0 ± 81.5	+25.3 (12.2, 38.4)**	9.7	19.2	+9.6 (1.7, 17.4)*
All	m	333	45.9 ± 28.3	29.4 ± 41.9	−16.5 (−12.1, −20.8)**	95.7 ± 76.7	108.8 ± 99.1	+13.1 (3.0, 23.2)**	10.2	17.2	+7.0 (1.8, 12.1)*
	f	385	41.8 ± 29.1	29.1 ± 34.3	−12.7 (−9.0, −16.3)**	76.8 ± 56.2	99.1 ± 82.1	+22.3 (14.8, 29.8)**	6.3	11.7	+5.3 (1.4, 8.4)*
	Ø	718	43.4 ± 28.9	29.3 ± 38.0	−14.4 (−11.6, −17.2)**	85.6 ± 67.1	103.6 ± 90.4	+18.0 (11.6, 24.2)**	8.1	14.2	+6.1 (2.9, 9.3)*

*n.* *s* non-significant, *m* male, *f* female, *Ø* mean of males and females, *M* mean, *s* standard deviation, *diff*. difference peri–pre, *95% CI* 95% confidence interval, *PA guideline adherence*: being physically active for 60 min or more a day during a week

*p < 0.05, **p < 0.01

The results show a significant increase in HA pre to peri of +46.8 min per day for the 6–10 year olds (*F*_*1, 612*_ = 49.97; *p* < 0.01) and +18.0 min for the 11–17 year olds (*F*_*1, 684*_ = 24.81; *p* < 0.01), paralleled by a decrease in SA of −10.8 (*F*_*1, 605*_ = 26.90; *p* < 0.01), respectively −14.4 (*F*_*1, 664*_ = 37.92; *p* < 0.01) minutes per day. This translates into an overall increase of the PA guideline adherence of +15.1% among the 6–10 year olds and +6.1% among the 11–17 year olds.

HA did not differ significantly between SES groups pre (6–10 year olds: *F*_*2, 623*_ = 1.91; *p* = 0.15; 11–17 year olds: *F*_*2, 712*_ = 1.30; *p* = 0.27) or peri (6–10 year olds: *F*_*2, 632*_ = 0.55; *p* = 0.58; 11–17 year olds: *F*_*2, 713*_ = 1.78; *p* = 0.17) lockdown. HA changes during the lockdown were not meaningfully affected by sex (6–10 year olds: *F*_*1, 632*_ = 0.07 *p* = 0.93; 11–17 year olds: *F*_*1, 734*_ = 2.16; *p* = 0.14) or SES group (6–10 year olds: *F*_*2, 621*_ = 0.71; *p* = 0.49; 11–17 year olds: *F*_*2, 70*__9_ = 1.13; *p* = 0.33). We also did not find any significant effect of sex (6–10 year olds: *F*_*1, 623*_ = 0.01; *p* = 0.09; 11–17 year olds: *F*_*1, 664*_ = 0.36; *p* = 0.85) and SES (6–10 year olds: *F*_*2, 614*_ = 2.81; *p* = 0.06; 11–17 year olds: *F*_*2, 689*_ = 1.52; *p* = 0.22) on the lockdown changes in SA (Table 2).

Overall, boys lost more SA than girls (6–10 year olds: −15.0 versus −10.1 min/day; 11–17 year olds: −16.5 versus −12.7 min/day) and girls increased their HA more than boys (6–10 year olds: +46.6 versus +47.2 min/day; 11–17 year olds: +13.1 versus +22.3 min/day). In sum, boys did still show more SA and HA, as well as a higher adherence to the guidelines peri lockdown compared to girls.

Table 3 shows different types of HA stratified by sex and selected environmental variables.

**Table 3:** Habitual physical activity before and during the COVID‑19 lockdown in Germany (MoMo study)

		Playing outside (minutes per day)		Walking and cycling (minutes per day)		Gardening (minutes per day)		Housework (minutes per day)		Total amount of habitual activity (minutes per day)		
	*N*	Pre (M)	Peri (M)	Pre (M)	Peri (M)	Pre (M)	Peri (M)	Pre (M)	Peri (M)	Pre (M ± s)	Peri (M ± s)	Peri–pre (diff. (95% CI))
*Sex*												
Male	686	55.6	73.2**	39.1	40.3 *n.* *s.*	8.5	16.3*	7.0	10.4*	109.1 ± 75.9	138.8 ± 109.5	+29.7 (21.9, 38.0)**
Female	701	43.6	58.9**	36.3	41.4 *n.* *s.*	5.0	12.1*	10.0	15.5**	93.7 ± 68.8	126.7 ± 100.0	+33.0 (26.3, 39.6)**
*Community size*												
<5000	278	52.7	69.4**	36.5	42.4**	11.9	21.8**	9.4	13.4**	108.6 ± 74.0	145.3 ± 113.2	+36.6 (25.2, 48.1)**
5000–<20,000	381	45.3	66.8**	37.1	41.4*	6.9	15.6**	10.3	13.2*	99.0 ± 74.0	136.7 ± 104.8	+37.7 (27.6, 47.8)**
20.000–<100,000	377	51.3	71.9**	37.3	39.8 *n.* *s.*	5.5	10.8**	7.3	13.2**	99.8 ± 70.4	133.0 ± 104.6	+33.2 (24.2, 42.3)**
100,000+	311	50.2	55.0 *n.* *s.*	40.0	40.1 *n.* *s.*	3.4	9.6**	7.0	12.0**	99.6 ± 73.0	115.9 ± 95.6	+16.4 (5.9, 26.8),**
*Housing situation*												
Multifamily home 6+ parties	146	52.9	50.8 *n.* *s.*	40.1	37.7 *n.* *s.*	3.7	10.0*	11.7	16.6	107.9 ± 88.9	115.1 ± 103.8	+7.2 (−8.3, 22.7) *n.* *s.*
Multifamily home	186	49.6	61.5*	37.4	45.4*	7.0	16.4**	9.2	14.3*	102.1 ± 83.0	134.1 ± 123.6	+32.0 (16.0, 48.0)**
Semi-detached house	274	44.4	62.4**	36.8	39.2 *n.* *s.*	4.8	8.0**	7.8	10.8*	92.8 ± 58.0	118.7 ± 93.1	+25.9 (15.7, 36.1)**
Detached house	739	50.6	71.5**	37.5	40.9**	8.0	16.66**	8.0	12.5**	102.5 ± 70.4	140.4 ± 103.0	+37.9 (31.1, 44.7)**
No garden/yard	260	50.7	47.2**	37.1	41.0 *n.* *s.*	3.4	10.2**	11.4	17.6**	104.6 ± 91.3	113.2 ± 112.6	+8.6 (−4.6, 21.9) *n.* *s.*
Garden/yard	1127	49.4	70.4**	40.2	40.3 *n.* *s.*	7.5	15.1**	7.9	11.9**	100.6 ± 67.9	137.1 ± 102.6	+36.5 (31.1, 42.0)**
*All participants*												
∑	1387	49.6	66.1**	37.7	40.9 *n.* *s.*	6.7	14.2**	8.5	13.0**	101.3 ± 72.8	132.7 ± 104.9	+31.3 (26.2, 36.4)**

*n.* *s* nonsignificant, *m* male, *f* female, *Ø* mean of males and females, *M* mean, *s* standard deviation, *95% CI* 95% confidence intervals

*/** Significant difference (*p < 0.05, **p < 0.01)

We found no overall significant differences in pre to peri HA changes between males and females (*F*_*1, 1368*_ = 0.41; *p* = 0.52). However, the size of the community did predict HA changes (*F*_*1, 1366*_ = 3.47; *p* = 0.02), mainly due to a lower increase of HA in communities with more than 100,000 inhabitants (Table 3).

Significant differences in pre to peri HA changes were also found for the type of building the participants live in (*F*_*3, 1364*_ = 4.56; *p* < 0.01). It is striking that children and adolescents living in multifamily homes with more than six parties did not increase their HA significantly, whereas children and adolescents living in detached houses increased their HA by an average of +37.9 min per day.

Lastly, the results show that living in a home with a garden/yard was very beneficial for increasing the HA during the lockdown. Whereas children and adolescents living in a home without a garden/yard showed only a non-significant numerical increase of +8.6 min HA per day, those living in a home with a garden/yard increased their HA significantly by an average of +36.5 min per day. Similar to the size of the community and the housing situation, the main effect of the lockdown showed a significant interaction with the fact whether the participants lived in a house with or without a garden/yard (*F*_*1, 1368*_ = 17.65; *p* < 0.01). This effect was especially pronounced in playing outside (*F*_*1, 1345*_ = 23.61; *p* < 0.01). Children and adolescents with access to a garden/yard increased their playing outside by 70.4 min per day compared to only 47.2 min per day without access.

With an effect of η^2^_p_ = 0.13 compared to 0.08 (size of the community) and 0.10 (housing situation), access to a garden was the most meaningful moderating variable on the increase in HA. Table 4 shows the amount of recreational ST pre and peri lockdown, as well as the adherence to ST guidelines stratified by age, SES, and sex.

**Table 4:** Recreational screen-time and guideline adherence by socioeconomic status before and during the COVID‑19 lockdown in Germany (MoMo study)

			Total amount of recreational ST (minutes per day)			ST guideline adherence (%)		
**Age** **6–10**		***N***	**Pre** **(M** **±** **s)**	**Peri** **(M** **±** **s)**	**Peri–pre** **(diff. (95% CI))**	**Pre** **(%)**	**Peri** **(%)**	**Peri–pre** **(diff. (95% CI))**
Low	m	44	134.1 ± 112.9	240.2 ± 150.7	+106.2 [61.3, 151.1]**	64.4	22.7	−41.7 [−57.5, −21.3]*
	f	45	127.0 ± 110.4	170.2 ± 110.3	+43.2 [17.3, 69.1]**	57.8	43.5	−14.3 [−33.1, +6.0] *n.* *s.*
	Ø	89	130.5 ± 111.1	204.8 ± 135.7	+74.3 [48.2, 100.5]**	61.1	33.3	−27.8 [−40.7, −13.2]*
Mid	m	200	87.9 ± 66.3	160.1 ± 101.4	+72.3 [60.1, 84.4]**	77.5	42.2	−35.3 [−43.8, −26.0]*
	f	159	85.8 ± 79.7	147.2 ± 116.6	+61.4[48.7, 74.1]**	81.8	53.1	−28.7 [−38.0, −18.6]*
	Ø	359	87.0 ± 72.4	154.4 ± 108.4	+67.4 [58.6, 76.2]**	79.4	47.0	−32.4 [−38.8, −25.6]*
High	m	91	68.6 ± 49.1	139.0 ± 97.3	+70.4 [55.9, 84.9]**	84.6	52.2	−32.4 [−44.2, −19.2]*
	f	87	63.2 ± 56.2	123.9 ± 82.6	+60.7 [48.9, 72.4]**	88.5	56.8	−31.7 [−43.3, −18.8]*
	Ø	178	66.0 ± 52.6	131.6 ± 90.5	+65.7 [56.3, 75.0]**	86.4	54.4	−32.1 [−40.5, −22.9]*
All	m	335	88.7 ± 72.4	165.2 ± 111.7	+76.5 [66.5, 86.4]**	77.6	42.2	−35.5 [−42.0, −28.4]*
	f	291	86.1 ± 82.1	143.2 ± 106.7	+57.2 [48.5, 65.8]**	79.7	52.6	−27.1 [−34.1, −19.6]*
	Ø	626	87.5 ± 77.0	154.9 ± 109.9	+67.5 [60.8, 74.2]**	78.6	47.1	−31.6 [−36.4, −26.4]*
**Age** **11–17**		***N***	**Pre** **(M** **±** **s)**	**Peri** **(M** **±** **s)**	**Peri-–pre** **(diff. (95% CI))**	**Pre** **(%)**	**Peri** **(%)**	**Peri–pre** **(diff. (95% CI))**
Low	m	61	285.3 ± 161.9	323.5 ± 160.5	+38.2 [−3.8, 80.1] *n.* *s.*	14.8	7.9	−6.8 [−18.7, +4.7] *n.* *s.*
	f	69	227.7 ± 145.1	271.0 ± 147.0	+43.2 [13.0, 73.4]**	25.7	12.9	−12.9 [−25.6, +0.3] *n.* *s.*
	Ø	130	254.8 ± 155.3	295.6 ± 155.1	+40.9 [15.8, 65.9]**	20.6	10.5	−10.1 [−18.8, −1.3]*
Mid	m	194	233.0 ± 144.3	312.1 ± 154.4	+79.0 [59.0, 99.1]**	25.3	10.3	−15.0 [−22.4, −7.5]*
	f	230	185.2 ± 121.2	255.1 ± 138.5	+70.0 [53.6, 86.3]**	36.1	15.8	−20.3 [−27.9, −12.4]*
	Ø	424	207.1 ± 134.3	281.2 ± 148.5	+74.1 [61.4, 86.8]**	31.1	13.3	−17.9 [−23.2, −12.3]*
High	m	71	176.7 ± 105.8	245.6 ± 119.8	+68.8 [43.5, 94.2]**	43.7	13.9	−29.8 [−42.9, −15.1]*
	f	84	151.2 ± 91.0	222.3 ± 129.9	+70.3 [45.1, 95.6]**	42.9	21.4	−21.4 [−34.4, −7.3]*
	Ø	155	163.3 ± 98.5	233.0 ± 125.5	+69.7 [51.9, 87.4]**	43.2	17.9	−25.3 [−34.7, −15.1]*
All	m		233.1 ± 145.0	299.7 ± 151.8	+66.6 [51.2, 82.0]**	26.5	11.3	−15.2 [−21.0, −9.4]*
	f		186.4 ± 123.7	249.1 ± 139.9	+62.7 [50.4, 75.1]**	35.4	16.9	−18.5 [−24.4, −12.5]*
	Ø		208.0 ± 135.9	272.5 ± 147.6	+64.5 [54.8, 74.2]**	31.3	14.3	−17.0 [−21.2, −12.8]*

*m* male, *f* female, *Ø* mean of males and females, *M* mean, *s* standard deviation, *diff.* difference peri–pre, *95% CI* 95% confidence interval, *ST guideline adherence*: Using recreational screen time media for 120 min or less a day during a week

*/** Significant difference (*p < 0.05, **p < 0.01)

We found an overall significant increase in recreational ST of +67.5 min per day for the 6–10 year olds (*F*_*1, 605*_ = 155.89; *p* < 0.01) and +64.5 min for the 11–17 year olds (*F*_*1, 681*_ = 75.83; *p* < 0.01). During the lockdown, the adherence with the ST guideline decreased significantly by −31.6% among the 6–10 year olds and −17.0% among the 11–17 year olds.

Recreational ST did differ significantly between SES groups pre (6–10 year olds: *F*_*2, 624*_ = 21.94; *p* < 0.01; 11–17 year olds: *F*_*2, 707*_ = 16.68; *p* = 0.01) and peri lockdown (6–10 year olds: *F*_*2, 633*_ = 13.68; *p* < 0.01; 11–17 year olds: *F*_*2, 715*_ = 8.37; *p* < 0.01). Among both age groups, only 19.7% of children from low SES families met the guidelines during the lockdown compared to 28.8% from mid-SES families and 37.5% from high-SES families.

Boys showed more recreational ST during the lockdown (6–10 year olds: *F*_*2, 644*_ = 7.67; *p* = 0.01; 11–17 year olds: *F*_*2, 744*_ = 21.34; *p* < 0.01) compared to girls. Pre lockdown, these differences were only significant for the 11–17 year olds (6–10 year olds: *F*_*2, 634*_ = 0.18; *p* = 0.68; 11–17 year olds: *F*_*2, 732*_ = 22.45; *p* < 0.01).

Changes in recreational ST were significantly affected by SES among the 11–17 year olds (*F*_*2, 706*_ = 3.24; *p* = 0.04) but not among the 6–10 year olds (*F*_*2, 623*_ = 3.24; *p* = 0.73). Boys showed a significantly higher increase in recreational ST compared to girls among the 6–10 year olds (*F*_*1, 615*_ = 6.18; *p* = 0.01) but not among the 11–17 year olds (*F*_*2, 681*_ = 0.30; *p* = 0.59).

## Discussion

Our study found a decrease of SA paralleled by an increase of habitual PA and recreational ST among children and adolescents living in Germany during the first COVID‑19 lockdown. These main results are in line with a study from Belgium (Constandt et al., 2020) but in contrast to data from other countries such as Canada (Guerrero et al., 2020; Moore et al., 2020), China (Xiang et al., 2020), and Italy (Pietrobelli et al., 2020). Overall, the behavioral changes led to an increase in the PA guideline adherence of +15.1% among the 6–10 year olds and +6.1% among the 11–17 year olds and to a decrease of the 2 h per day ST guideline adherence of −31.6% and −17.0%, respectively.

Since it is known that PA behavior is particularly affected by socioeconomic variables (Eime, Harvey, Craike, Symons, & Payne, 2013; Post et al., 2018; de Boer, Dekker, Koning, Navis, & Mierau, 2020), we focused on the role of potential PA correlates to identify potential subgroups of youth with peculiar behavior during the first lockdown in the present analyses. Children, as well as adolescents, and particularly girls from families with low SES did show lower amounts of SA and a lower guideline adherence pre, but not peri lockdown (Table 2). Studies all over the world found that socioeconomic and sociocultural differences in PA behavior among youth are especially pronounced in organized forms of sports (Eime et al., 2013; Post et al., 2018; de Boer et al., 2020; Schmidt et al., 2020a). Since all forms of organized sports were forbidden by governmental law during the first lockdown and therefore, entry barriers to organized SA played no role for PA behavior, we experienced a harmonization of PA guideline adherence between SES groups as well as boys and girls peri lockdown. These insights underline the importance of structural barriers for PA and may be useful in tailoring future PA interventions that target youth from families with lower SES in particular.

As organized SA was no option for youth during the lockdown, HA and especially unstructured playing outside became more important for children (+46.8 min HA per week) and adolescents (+18.0 min HA per week). This shift from structured to unstructured activities with a focus on outdoor play is in line with studies from countries that allowed exercising outdoors during the first lockdown, for example in the US (Dunton et al., 2020), but in contrast to most studies that focus primarily on adults (Mutz & Gerke, 2020). These circumstances speak for the theory that insufficient levels of PA among youth are partly context-driven (e.g., too much forced sitting during school and homework). To understand and classify these changes, studies that analyze the impact of a higher proportion of unstructured activities on overall PA intensity and children’s and adolescents’ health and fitness are needed.

We stated that the fact that children and adolescents in Germany spent more time active peri than post lockdown may be explained by short-term effects of simply having more recreational time to do so, but also theoretically by self-determination theory and a more pronounced focus on health (Schmidt et al., 2020b). The analyses on the influence of the SES and the individual housing situation (Table 3) showed that the increase in HA and outdoor play was not ultimately generalizable. We found no significant effects of sex and SES on the changes in HA pre and peri lockdown. However, we did find meaningful influences of the community size in favor of neighborhoods in towns and cities smaller than 100,000 inhabitants and in favor of homes with less than six parties. Playing outside, walking, cycling, as well as gardening increased significantly more among youth living in those environments. Interestingly, access to a garden was the most meaningful predictor of the lockdown changes in HA and, thus, remaining sufficient levels of PA during the lockdown. In our first study, we concluded that being able to go outdoors safely with rules of social distancing and contact to at least one known person was the main explanation for the differences in youth PA levels during the different lockdowns all over the world (Schmidt et al., 2020b) with many countries reporting an overall decrease of PA among youth (Guerrero et al., 2020; Moore et al., 2020; Xiang et al., 2020; Pietrobelli et al., 2020). The present results confirm this hypothesis but add that this behavior is not only affected by the permission to do so, but also by an activity-friendly environment that enables it. We also stated that advanced augmented reality and virtual reality solutions may be a way to enable exercising and playing with friends throughout all socioeconomic classes in highly populated areas such as metropolises or non-activity-friendly climate zones in the future (Schmidt et al., 2020b). However, as long as such options are not available, the present results highly emphasize the demand for appealing social and natural facilities that allow exercising and playing outside to everyone, independent of the size of the community or population density. Interventions on the change of behavior among inactive children and adolescents such as cooperations between schools and sports clubs or the offering of extracurricular sports activities offer another possibility to enhance PA among youth. However, structured opportunities inherit social barriers (Will, Schmidt, & Woll, 2016) and may be significantly less needed if the environment itself offers inspiring possibilities to be active.

Recreational ST was considerably lower in higher SES groups among both age groups pre-lockdown which is in line with recent studies from most western countries (Gebremariam, Henjum, Terragni, & Torheim, 2020). However, this trend is not (yet) generalizable all over the world. For example, a recent study found that in Iran, individuals with higher SES had a significantly higher risk of prolonged ST, watching TV, and working with computers (Heshmat et al., 2018). Earlier studies show that this is similar to the behavior in western countries during the early 2000s (Vandelanotte, Sugiyama, Gardiner, & Owen, 2009). These circumstances were summarized before in a meta-analysis by Mielke, Brown, Nunes, Silva, and Hallal (2017) which found that the direction of the association between SES and sedentary behaviors varies between high-income and low- to middle-income countries. Besides that, the majority of studies with data from the early to mid-2010s and before found that girls have a decreased risk of prolonged ST compared to boys (Heshmat et al., 2018; Sigmundová et al., 2017; Saunders & Vallance, 2017). However, this trend has to be questioned with recent data as more and more studies find similar ST among girls and boys, most likely explained by an increase in the use of social networking platforms by girls (Hinkley, Brown, Carson, & Teychenne, 2018; Simón-Montañes, Solana, García-Gonzalez, Catalán, & Sevil-Serrano, 2019).

Peri lockdown, ST between SES groups differed significantly among the 6–10 year olds but not among the 11–17 year olds, where youth from higher SES groups showed only a numerical, but not a statistically significant higher increase of ST peri lockdown. The overall increase in ST of about an hour in both age groups peri lockdown is striking and led to a meaningful decrease in reaching the ST guidelines among each age and SES group. Other studies that examined ST of youth during the first lockdown in Germany (Langmeyer, Guglhör-Rudan, Naab, Urlen, & Winklhofer, 2020) and China (Xiang et al., 2020) confirmed this trend.

While correlational studies have shown that children who exceed screen time recommendations score lower on cognitive assessments scores (Walsh et al., 2018) and a combination of screen time and too little sleep has been associated with heightened impulsivity among 8–11 year olds (Guerrero et al., 2019), screen-time recommendations have been criticized lately as evidence for an absolute screen time limit based on systematic reviews is weak (Stiglic & Viner, 2019; Przybylski & Weinstein, 2019). The evidence underlying the perception of harmful ST is limited and often clouded by confounding factors including socioeconomic grouping and negative associated behaviors such as snacking and reduced interest in PA (Stiglic & Viner, 2019; Przybylski & Weinstein, 2019). It was found that current policy statements tend to be more definitive than is warranted by the underlying science, and often ignore conflicting research results (Elson et al., 2019). Due to rapid changes in the spectrum of on- and offline ST activities, the idea of ST as a one-dimensional activity has changed, and researchers all over the world are recognizing that not all ST is created equal (Ashton & Beattie, 2019). Growing up digital becomes more and more important for occupation and career and even health and quality of life in later stages of life. For example, a recent study showed that smartphone non-users showed worse mental health, lower perceived quality of life, more sedentarism, and a greater tendency towards being overweight/obese as well as a higher feeling of loneliness (Pedrero-Pérez et al., 2019) compared to smartphone users. Therefore, more and more scientists demand a paradigm shift, away from pragmatic advice and towards an approach that is more tailored to the individual, including practical techniques for healthy and sensible strategies for managing ST, instead of just forbid it (Ashton & Beattie, 2019; Viner et al., 2019). The WHO recommendations that were published after the conduction of this study accordingly backed away from 120 min and used the term “limit the amount of time spent being sedentary, particularly the amount of recreational screen time” (Bull et al., 2020). We also strongly recommend focusing on an individual “why”, instead of “how long” when recommending an appropriate amount of recreational ST to youth and therefore do not interpret our results as a strong argument towards the need for a general reduction of ST, that is irrespective of its content.

Although we were able to fall back on nationwide data pre and peri lockdown within the framework of MoMo, there are some limitations to this study. First, the representativeness of our longitudinal sample is limited because of the unforeseen COVID‑19 outbreak during the collection of the representative pre-study sample. Out of a total of 167 sample points across Germany, we reached only 114 before we had to interrupt the field research during the pre-study. However, MoMo includes a circle concept that ensures that sample points from each region in Germany are reached every study year, i.e., at least twice during one wave. This ensures that sample points with different environmental contexts such as the population of the residential area and region within Germany are tested during different months of the year. Since more than one year and one full circle was completed before the lockdown, we were able to gather data from all regions within Germany, and the reduction in representativeness because of missing sample points can be considered as small.

Second, our results are based on questionnaires rather than device-based measures because we decided that it is not ethically justifiable to use accelerometry on a large sample during the first lockdown where we had very limited information about the virus. Overall, device-based studies of PA during the lockdown are scarce and the little data that have been published so far rely on nonrepresentative ad hoc samples (Hemphil et al., 2020) or already active people (Garmin, 2020). In addition, they lack methodological comparability, especially when ST is also addressed. The largest sources of published device-based measured PA peri lockdown are country-stratified data from Garmin wearables (Garmin, 2020) and a smartphone app (Tison et al., 2020). The Garmin data show that different activities such as walking or outdoor and indoor cycling changed differently among countries, with most of them increasing (Garmin, 2020). The study from Tison and colleagues analyzed step counts and confirmed substantial differences between countries but reported decreases in step counts within 30 days of the pandemic from 6.9% in Sweden to 48.7% in Italy (Tison et al., 2020). However, the use of a questionnaire has also important advantages over device-based measurements as the setting and type of the activity can be tracked more easily (Nigg et al., 2020). To utilize this, our questionnaire has been tailored to the different PA settings in Germany (Schmidt et al., 2016).

Third, as our pre to peri lockdown comparison forms a natural experiment, there is no control group and we can only assume that the lockdown was causal for the changes in PA and ST behavior. The weather was untypically warm during April 2020 in Germany with a mean temperature of 10.4 °C with and an average of 292.4 sunshine hours compared to 9.6 °C and 227.9 h in 2019 (German Weather Service, 2019; German Weather Service, 2020). Future data from the second lockdown will help to evaluate the effects.

Fourth, when interpreting our results, one should keep in mind that the mean age of our participants peri lockdown exceeded the mean age pre lockdown by one year. Since studies show that especially nonorganized PA declines during maturation (Tremblay et al., 2016; Schmidt et al., 2020a; Armstrong & Welsman, 2006; Ingram, 2000), this may have led to a small underestimation of the observed increase in PA in our study.

Lastly, we did rerun our rm(repeated measurements)ANOVAs with the metrical sum score instead of the categorized three-level SES and compared the f‑ and *p*-values with those reported in the results section. As expected, f‑values changed slightly when using the whole variance of the SES but we did not find any shift in significance for the reported within- and between-effects of SES on neither SA, HA, nor ST.

## Conclusion

Our study showed that not every household had the opportunity to allow and/or encourage its children to play outside during the first COVID‑19 lockdown in Germany and recreational ST significantly increased by approximately one hour per day. To prevent the development of disadvantaged subgroups during potential future lockdowns and thereafter, restriction policies, communities, and parents need to enable access to nonorganized PA to all children and adolescents at all times. This may be achieved by providing sophisticated information about the importance of PA and outdoor play for parents and by working on opportunities for being active safely outside by local authorities. The fact that, despite these unnatural, PA-unfriendly circumstances, the compliance with the recommended 60 min of daily PA increased after having been stable for two decades (Bucksch, Inchley, Hamrik, Finne, & Kolip, 2014; Schmidt et al., 2020a) is striking and needs to be analyzed further. With the availability of future, representative, cross-sectional samples and with the data gathered during the second lockdown (Schmidt et al., 2021), we will be able to make more precise assumptions about the stability of these changes and the overall impact of the COVID‑19 pandemic on PA behavior.

## Declarations

All procedures performed in studies involving human participants or on human tissue were in accordance with the ethical standards of the institutional and/or national research committee and with the 1975 Helsinki declaration and its later amendments or comparable ethical standards. For MoMo, ethics approval was obtained from the Charité Universitätsmedizin Berlin ethics committee, the University of Konstanz, and the ethics committee of the Karlsruhe Institute of Technology. The Federal Commissioner for Data Protection and Freedom of Information was informed about the study and approved it. Informed consent was obtained from all individual participants included in the study.
